# Microstructure and Mechanical Properties of TaNbVTiAl_x_ Refractory High-Entropy Alloys

**DOI:** 10.3390/e22030282

**Published:** 2020-02-29

**Authors:** Li Xiang, Wenmin Guo, Bin Liu, Ao Fu, Jianbo Li, Qihong Fang, Yong Liu

**Affiliations:** 1 State Key Lab of Powder Metallurgy, Central South University, Changsha 410083, China; xl1536417167@163.com (L.X.); wenminguo@hotmail.com (W.G.); iceiceice_isu@hotmail.com (A.F.); yonliu@csu.edu.cn (Y.L.); 2 School of Materials Science and Engineering, Chongqing University, Chongqing 400044, China; lijianbo1205@163.com; 3 College of Mechanical and Vehicle Engineering, Hunan University, Changsha 410082, China

**Keywords:** high-entropy alloy, refractory alloy, powder metallurgy, specific strength, ductile

## Abstract

A series of TaNbVTiAl_x_ (x = 0, 0.2, 0.4, 0.6, 0.8, and 1.0) refractory high-entropy alloys (RHEAs) with high specific strength and reasonable plasticity were prepared using powder metallurgy (P/M) technology. This paper studied their microstructure and compression properties. The results show that all the TaNbVTiAl_x_ RHEAs exhibited a single BCC solid solution microstructure with no elemental segregation. The P/M TaNbVTiAl_x_ RHEAs showed excellent room-temperature specific strength (207.11 MPa*cm^3^/g) and high-temperature specific strength (88.37 MPa*cm^3^/g at 900 °C and 16.03 MPa*cm^3^/g at 1200 °C), with reasonable plasticity, suggesting that these RHEAs have potential to be applied at temperatures >1200 °C. The reasons for the excellent mechanical properties of P/M TaNbVTiAl_0.2_ RHEA were the uniform microstructure and solid solution strengthening effect.

## 1. Introduction

With the development of the aerospace and nuclear industries, the service temperature of traditional nickel-base superalloys is close to its melting point [1]. Therefore, the development of new refractory alloys should be accelerated. In the last decade, refractory high-entropy alloys (RHEAs), which contain five or more refractory elements with a concentration between 5 at.% and 35 at.% [2], have attracted much attention due to their high melting points and outstanding high-temperature mechanical properties. Studies have shown that at 1600 °C, NbMoTaW RHEA has a higher yield strength than the Inconel 718 and Haynes 230 alloys, which can reach 405 MPa [3]. Deriving from this alloying design strategy, a series of RHEAs have been developed, such as MoNbTaV [4], VNbMoTaW [3], and NbMoTaWTi [5]. These RHEAs are considered backup alloys for high-temperature structural materials. However, such alloys generally exhibit lower plasticity and poor machinability, making them unusable for industrial applications. 

Many researchers have done a lot of work to improve the processability of RHEAs [6,7,8]. Chen et al. [9] reported that the plasticity of RHEAs can be improved by reducing the number of valence electrons in a single-phase BCC solid solution. Sheikh et al. [10] reported a new RHEA (Hf_0.5_Nb_0.5_Ta_0.5_Ti_1.5_Zr) which achieved fracture strength of 1000 MPa and fracture plasticity of 20%. Guo et al. [11] prepared a novel NbTaTiV RHEA through powder metallurgy (P/M) method, which has a yield strength of 1.37 GPa and a fracture strain of 23%. Unfortunately, although these RHEAs have high strength and acceptable plastic strain, their density is still too high, which hinders the practical application. 

The literature [12,13,14] shows that adding light elements (such as Al) to RHEAs can effectively reduce the density and increase the specific strength of the alloys. For example, Senkov et al. [12] prepared Al_0.4_Hf_0.6_NbTaTiZr RHEA by adding Al into the HfNbTaTiZr RHEA. The density of the alloy decreased from 9.94 g/cm^3^ to 9.05 g/cm^3^, and the specific yield strength increased from 93.56 MPa*cm^3^/g to 203.43 MPa*cm^3^/g. Yurchenko et al. [13] reported that with the increase of Al content, the density of CrNbTiVZrAl_x_ RHEA gradually decreased from 6.56 g/cm^3^ (CrNbTiVZr) to 6.21 g/cm^3^ (Al_0.5_CrNbTiVZr), and the specific strength increased from 192.07 MPa*cm^3^/g to 262.48 MPa*cm^3^/g. Xu et al. [14] reduced the density of the Ti_50-x_Al_x_V_20_Nb_20_Mo_10_ RHEA from 6.102 g/cm^3^ (Ti_40_Al_10_V_20_Nb_20_Mo_10_) to 5.876 g/cm^3^ (Ti_30_Al_20_V_20_Nb_20_Mo_10_) by adjusting the Al content, and the room-temperature specific yield strength increased from 147.49 MPa*cm^3^/g to 202.01 MPa*cm^3^/g. Although the density and specific strength of these alloys are significantly improved after the addition of aluminum, their plasticity is generally reduced. Therefore, choosing a refractory high-entropy alloy with both high strength and high plasticity as a matrix, and then reducing its density by adding light elements such as Al, is an effective method to obtain structural materials with high strength, low density, and good plasticity. The recently reported TaNbVTi alloy meets the requirements of the matrix very well [11]. Furthermore, Yang et al. [15] reported that the arc-melted NbTiVTaAl_0.25_ HEA has a low density of 8.78 g/cm^3^ and reasonable plasticity greater than 50%. Nevertheless, the yield strength of this alloy is only 1330 MPa (specific strength of 151.48 MPa*cm^3^/g), which has the possibility of further improvement compared with most refractory high-entropy alloys.

Powder metallurgy is one of the effective methods for preparing refractory high-entropy alloys. Due to the multicomponents in the RHEAs and the large differences in melting point, coarse dendrites and severe segregation are easily formed during the melting process, resulting in a multiphase structure with even several intermetallic phases [16]. Studies [6,7,11,16,17] have shown that a P/M method is one of the effective methods for preparing multicomponent RHEAs, which can avoid component segregation and obtain a uniform equiaxed crystal structure. The mechanical properties of these RHEAs are also expected to be further improved because the P/M technology uses a powder mixing method to make the composition uniform. Moreover, spark plasma sintering (SPS) can make the powder completely solidified in a few minutes, which can effectively inhibit the grain growth and composition segregation. Therefore, a multicomponent alloy without segregation and fine grains can be obtained. In addition, considering the poor machinability of RHEAs, the advantages of the near-net forming technology of P/M technology can significantly broaden the application range. In this study, we developed a series of Al-alloyed RHEAs (TaNbVTiAl_x_) through a P/M method. The effect of Al on the microstructure and mechanical properties of the TaNbVTiAl_x_ RHEAs was investigated. The strengthening mechanism was also discussed.

## 2. Materials and Methods 

TaNbVTiAl_x_ (x = 0, 0.2, 0.4, 0.6, 0.8, and 1.0) RHEAs, which are called Al0, Al0.2, Al0.4, Al0.6, Al0.8, and Al1.0, were prepared using an elemental P/M method. The theoretical compositions of the alloys are shown in Table 1.

**Figure 1:**
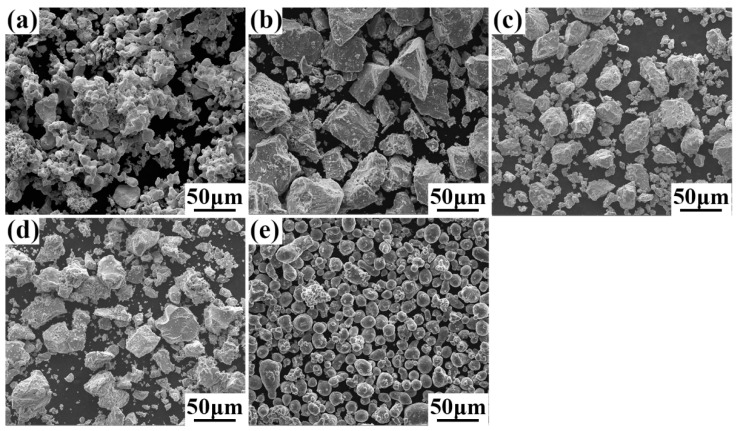
Morphologies of the raw powders. (**a**) Ta, (**b**) Nb, (**c**) V, (**d**) Ti, and (**e**) Al.

Figure 1 illustrates the macroscopic morphology of elemental Ta, Nb, V, Ti, and Al powders. The average particle size and impurity content are presented in Table 2. The Ta, Nb, V, and Ti powders have irregular shapes and the powder sizes are not larger than 30 μm. The Al powder has a nearly spherical shape with particle size less than 20 μm.

The raw powders (purity > 99.5 wt.%) with different compositions were mixed and ball-milled in a stainless steel ball mill tank. The mass ratio of stainless steel ball: powder was 10:1 and the ball mill was operated at 150 rpm for 8 h. The milled powders were then sintered by using SPS (D25/3, FCT, Rauenstein, Germany). The powders were heated to 700 °C without pressure, and then the temperature was further increased to 1700 °C by applying pressure of 30 MPa; the holding time was 10 min. The heating rate during the entire sintering process was 100 °C/min. Throughout the sintering process, the high-purity argon gas charged into the furnace as a protective atmosphere to prevent oxidation.

Test specimens (d 6 × 9 mm^3^) for room-temperature compression and high-temperature compression were cut from the sintered RHEAs by using an electrical discharge machining (EDM) method. An INSTRON-5569 test system was used for room-temperature compression experiments with a compression rate of 10^−3^ s^−1^. High-temperature compression experiments were carried out with a Gleeble-3180 device with a strain rate of 10^−3^ s^−1^ at 900 °C, 1000 °C, 1100 °C, and 1200 °C, respectively. 

Particle size distributions of the powders were measured by a laser particle size analyzer (Mastersizer 3000, Malvern, UK). The impurity elements in the powders were detected by an elemental analyzer (TCH600, LECO, San Jose, America). Phase analyses were performed by Cu-Ka target X-ray diffractometer (XRD, D/max 2500, CORP, Tokyo, Japan). Microstructures were observed via electron microscopy (SEM, G3-US, FEI, Hillsboro, America) with an electron backscatter diffraction (EBSD) device. A field emission probe microscope (EPMA, JXA-8530F, JEOL, Tokyo, Japan) was used to characterize the element distribution of the samples.

## 3. Results

### 3.1. Microstructures of the TaNbVTiAl_x_ RHEAs

**Figure 2:**
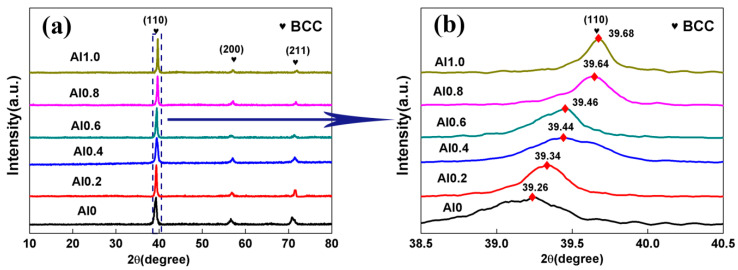
(**a**) XRD patterns of the TaNbVTiAl_x_ RHEAs; (**b**) the enlarged (110) peaks.

Figure 2 shows the XRD patterns of the TaNbVTiAl_x_ RHEAs. From Figure 2a, it can be seen that the TaNbVTiAl_x_ RHEAs had three diffraction peaks, which corresponded to the (110), (200), and (211) crystal planes, respectively. The positions of these crystal planes were consistent with those of the diffraction peaks of a BCC single phase. Therefore, all the TaNbVTiAl_x_ RHEAs exhibited a typical BCC solid solution microstructure. As shown in Figure 2b, the diffraction peak of the (110) shifted significantly from 39.26° to 39.68° as the Al content increased. It means that the (110) crystal plane lattice parameter gradually reduced with the increase of the Al content. Table 3 lists the crystal structures, calculated lattice parameters, theoretical lattice parameters, calculated melting temperatures, and theoretical densities of these alloys. The calculated lattice parameters were obtained based on the XRD results by a formula as follows [18]:

(1) 2dsinθ=λ,

(2) $$d=a/\sqrt{h^{2}+k^{2}+l^{2}},$$

(3) $$a=\lambda \sqrt{h^{2}+k^{2}+l^{2}}/\left(2\sin\theta \right),\;$$

where d is the crystal surface spacing; θ is the diffraction angle; *λ* is the X-ray diffraction wavelength (*λ* = 1.5406 Å); a is the lattice constant; h, k, l are the crystal surface index. 

In addition, the theoretical lattice parameter of these RHEAs can be calculated by the element’s theoretical lattice parameter of the RHEA. The calculation formula is as follows [19]:

(4) $$a_{\mathrm{mix}}=\sum_{i=1}^{n}c_{i}a_{i},$$

where c_i_ is the content of the i component (at.%); a_i_ is the lattice constant of the i component. 

As seen in Table 3, with an increase of Al content, the calculated lattice parameter decreased from 3.243 Å to 3.210 Å, while the theoretical lattice parameter increased from 3.230 Å to 3.394 Å. The reason for the difference is that the p-layer saturated electron orbit of Al atom was easy to hybridize with the d-layer unsaturated electron orbit of transition metal to form a covalent bond, while the length of the covalent bond was small [20]. Therefore, with the increase of Al content, the hybrid effect was more obvious, and the lattice constant decreased. The atomic radius of Al is smaller than that of Ta, Nb, and Ti, and obviously larger than that of V. Moreover, the single-phase BCC solid solution structure was always present in these alloys, which indicated that Al could easily form a solid solution with the other elements, and enhance the high-entropy effect of the alloy. Therefore, the increase of the Al element led to the increase of lattice distortion, and eventually, led to the difference between calculated lattice parameters and theoretical lattice parameters. Similar results have been reported in NbTiMoVAl_x_ RHEAs [18]. 

The theoretical densities of TaNbVTiAl_x_ RHEAs were calculated by the following formula [21]:

(5) $$\rho =\sum c_{i\;}M_{i}/\sum \frac{c_{i}M_{i}}{\rho_{i}},$$

where the c_i_, M_i_, and ρ_i_ represent the concentration, molar mass, and theoretical density of the i component, respectively. 

As the Al content increased, the density of the TaNbVTiAl_x_ RHEAs decreased gradually from 9.16 g/cm^3^ of Al0 alloy to 7.89 g/cm^3^ of Al1.0 alloy, which was very close to that of nickel-based superalloys [22].

**Figure 3:**
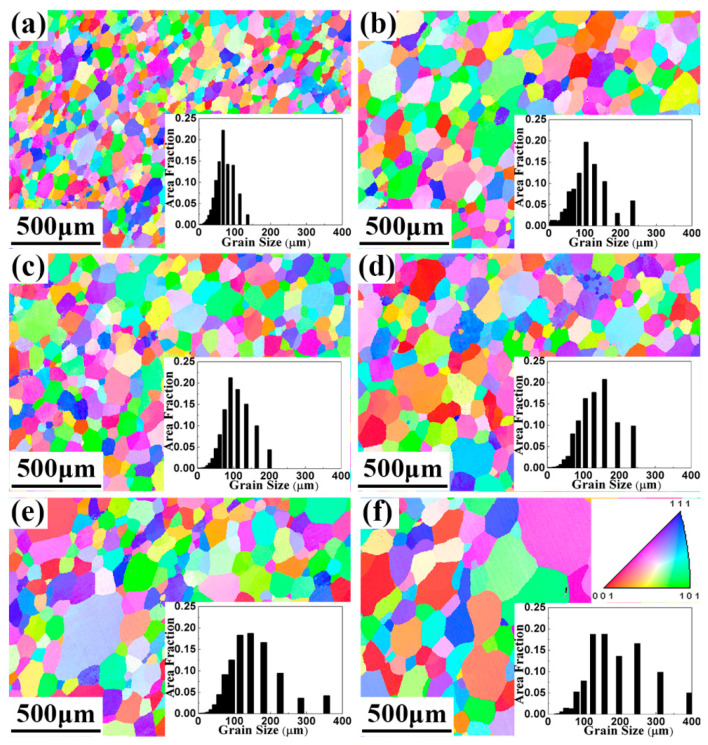
Inverse pole figure (IPF) and grain size distribution maps of the TaNbVTiAl_x_ RHEAs: (**a**) Al0, (**b**) Al0.2, (**c**) Al0.4, (**d**) Al0.6, (**e**) Al0.8, and (**f**) Al1.0.

**Figure 4:**
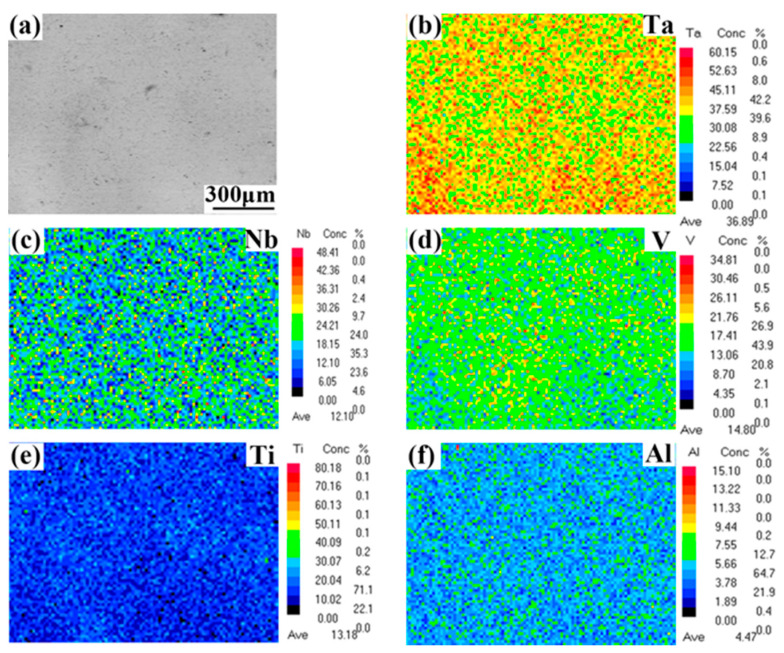
**Electron probe microanalysis** (EPMA) results of the sintered Al1.0 RHEA: (**a**) SEM image, (**b**) Ta, (**c**) Nb, (**d**) V, (**e**) Ti, and (**f**) Al.

Figure 3 illustrates the inverse pole figure (IPF) and grain distribution maps of the TaNbVTiAl_x_ RHEAs in the transverse direction. The average grain sizes were 69, 101, 106, 135, 147, and 187 μm for the Al0, Al0.2, Al0.4, Al0.6, Al08, and Al1.0, respectively. The results indicated that the higher the Al atom ratio, the larger the grain size of TaNbVTiAl_x_ RHEAs, which may be because of the higher the atomic ratio of aluminum, the lower the melting point of the alloy. At the same sintering temperature, the lower the melting point of the alloy, the closer it was to the sintering temperature and the faster the grain growth rate, resulting in an increase of grain size. In addition, there were no obvious textures in the as-sintered RHEAs, as shown in Figure 3. Figure 4 shows the EPMA results of the TaNbVTiAl_1.0_ RHEA. It can be found that the distribution map of all elements shows a single color without obvious bright spots. Therefore, the distribution of all elements was uniform and no significant segregation was observed.

### 3.2. Mechanical Properties of the TaNbVTiAl_x_ RHEAs

**Figure 5:**
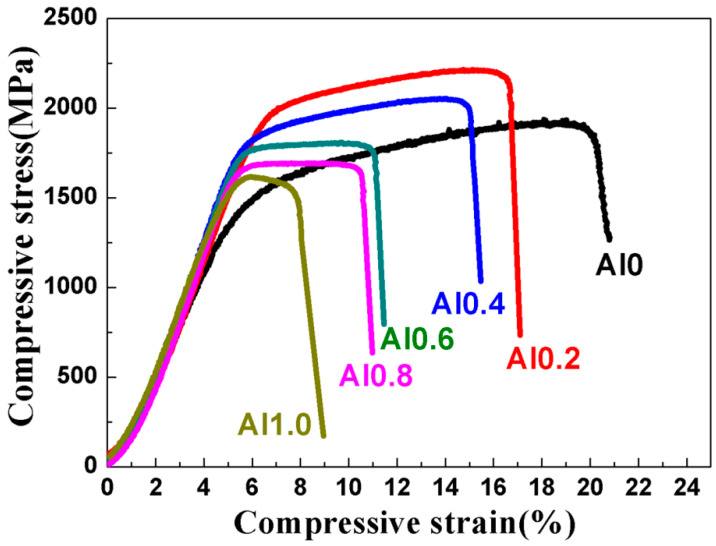
Room-temperature compressive stress–strain curves of the TaNbVTiAl_x_ RHEAs.

**Figure 6:**
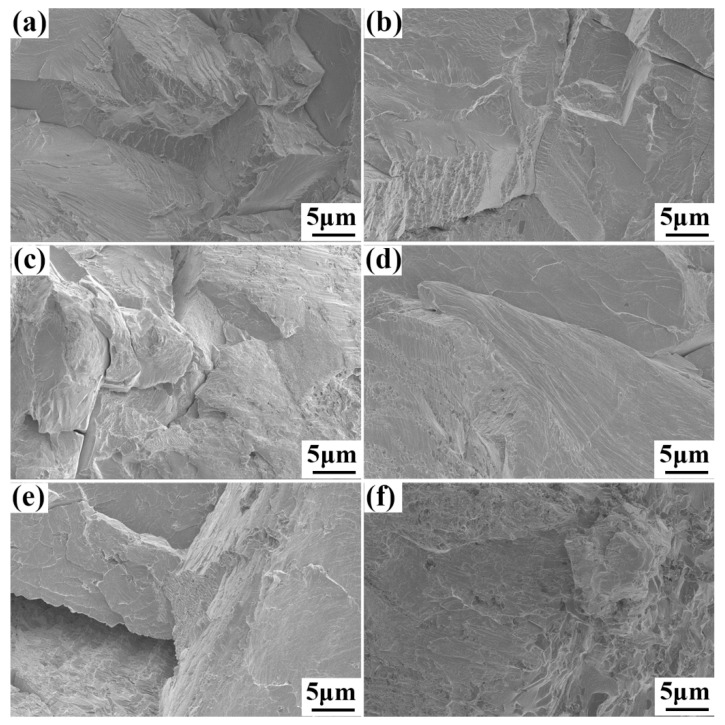
Fracture morphologies of the TaNbVTiAl_x_ RHEAs at room temperature: (**a**) Al0, (**b**) Al0.2, (**c**) Al0.4, (**d**) Al0.6, (**e**) Al0.8, and (**f**) Al1.0.

Figure 5 illustrates the room-temperature compressive stress–strain curves of the TaNbVTiAl_x_ RHEAs. The yield strength, compressive strength, and plastic strain of the Al0 alloy were 1391 MPa, 1932 MPa, and 15%, respectively. With an increase in the Al content to Al0.2, the yield strength and compressive strength increased to 1835 MPa and 2217 MPa, indicating that the addition of Al alloy elements significantly improved the strength, while the compressive strain decreased to about 10%. With the further increase of Al content, a gradual decrease in compressive strength occurred, while the plasticity continued to decrease. When x = 1.0, the yield strength, compressive strength, and plastic strain decreased to only 1450 MPa, 1619 MPa, and 2.5%, respectively. Table 4 lists the mechanical properties of the typical RHEAs reported in the literature and this work. It is shown that the Al0.2 RHEA had the highest specific yield strength, which is higher than most of the RHEAs, such as NbMoTaW [3], TaNbHfZr [23], TiZrNbTa [19], TaNbHfZrTi [24], Al_0.4_Hf_0.6_NbTaTiZr [12], and Al_0.21_HfNbTiZr [25] RHEAs, with reasonable compressive plasticity of about 10%. Compared with NbTiVTaAl_x_ [15] prepared by arc melting, the specific strength was greatly improved. Figure 6 shows the morphologies of the fracture surface of the TaNbVTiAl_x_ RHEAs. The fracture surfaces of all the RHEAs show classic rivers and step shapes, suggesting that the fracture mode was a typical brittle cleavage fracture.

**Figure 7:**
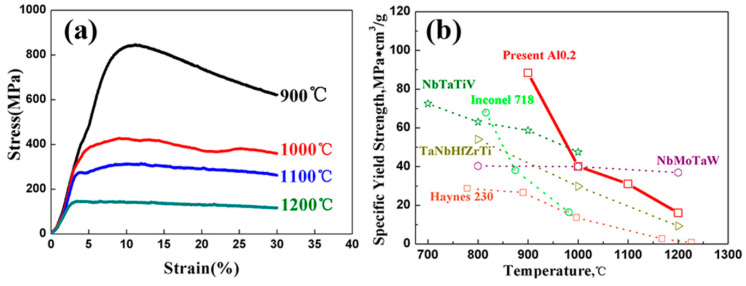
(**a**) High-temperature compressive stress–strain curves of Al0.2 RHEA at 900 °C–1200 °C and (**b**) comparison of high-temperature mechanical properties of several typical high-temperature alloys and the present Al0.2 RHEA.

Figure 7 shows the high-temperature compressive properties of the Al0.2 RHEA. It is shown that the Al0.2 alloy had a yield strength of 783 MPa at 900 °C (specific yield strength was about 88.37 MPa*cm^3^/g). As can be seen from Figure 7b, the high-temperature (<1000 °C) specific strength was better than that of the typical NbMoTaW RHEA [3], TaNbHfZrTi RHEA [26], NbTaTiV RHEA [11], Ni-based IN718 alloy, and Haynes 230 alloy [3]. This is because the single-phase BCC structure with multiple components and melting elements had slower element diffusion at higher temperatures [27]. Therefore, the high-temperature softening resistance of the alloy could be improved. At 1200 °C, the Al0.2 RHEA still kept a yield strength of 142 MPa, suggesting that the material has the possibility for use at high temperatures (>1200 °C).

## 4. Discussion

### 4.1. Phase Prediction

Predicting the phase structure of high-entropy alloys (HEAs) is a challenging task. At present, researchers have found a semiempirical method to determine the generation of a solid solution in HEAs [28]. According to the literature, the main factors affecting structures are as follows: the enthalpy of mixing (−15 kJ/mol ≤ ΔHmix ≤ 5 kJ/mol), radius asymmetry (δ < 6.6%) and entropy/enthalpy ratio (Ω > 1.1) [29,30]. Studies have [31,32] calculated the valence electron concentration (VEC) values through the relevant parameters of the HEAs, thereby obtaining the structure type of the solid solution. When the VEC ≤ 6.87, the HEAs generally exhibit BCC solid solution structure. If the VEC is between 6.78 and 8, they usually show a BCC + FCC two-phase solid solution structure. While when the VEC ≥ 8, the HEAs are mainly FCC solid solution phase. The calculation formula for the main parameters is as follows [33]:

$$\mathrm{Ideal}\;\mathrm{entropy}\;\mathrm{of}\;\mathrm{mixing},\;{\Delta S}_{\mathrm{mix}}=-R\sum_{i=1}^{N}c_{i}{\mathrm{lnc}}_{i}$$

$$\mathrm{Enthalpy}\;\mathrm{of}\;\mathrm{mixing},\;{\Delta H}_{\mathrm{mix}}=4\sum_{i=1,j\neq i}^{N}{\left({\Delta H}_{\mathrm{mix}}\right)}_{\mathrm{ij}}c_{i}c_{j}$$

$$\mathrm{Mean}\;\mathrm{melting}\;\mathrm{temperature},\;T_{m}=\sum_{i=1}^{N}c_{i}{\left(T_{m}\right)}_{i}$$

$$\mathrm{Entropy}/\mathrm{enthalpy}\;\mathrm{ratio},\;\Omega =\frac{T_{m}{\Delta S}_{\mathrm{mix}}}{\left|{\Delta H}_{\mathrm{mix}}\right|}$$

$$\mathrm{Valence}\;\mathrm{electron}\;\mathrm{concentration},\;\mathrm{VEC}=\sum_{i=1}^{N}c_{i}{\mathrm{VEC}}_{i}$$

$$\mathrm{Radius}\;\mathrm{asymmetry},\;\delta =\sqrt{\sum_{i=1}^{N}c_{i}{\left(1-\frac{r_{i}}{\;\bar{r}}\right)}^{2}}$$

where i and j represent different component elements; R is expressed as the gas constant (8.314 J/(K*mol)); c_i_ and c_j_ are the atomic fraction of the i and j component in the alloy; (ΔH_mix_)ij is the enthalpy of mixing of the two elements i and j [34]; (Tm)i is the theoretical melting temperature of the i component element; VECi is the valence electron concentration of the i component element [31]. 

Referring to the above semiempirical criteria, the calculation results of TaNbVTiAl_x_ RHEAs are shown in Table 5. It is shown that all the TaNbVTiAl_x_ RHEAs were located in the region of BCC solid solution. The prediction results were basically consistent with the conclusions of this study.

### 4.2. Strengthening Mechanism

The TaNbVTiAl_x_ RHEAs prepared by a P/M method exhibited high specific strength and reasonable plasticity both at room temperature and high temperature. The high yield strength of the RHEAs may have come from the uniform microstructure and the solid solution strengthening effect, while the reasonable plasticity may have resulted from the ductile TaNbVTi matrix, which has similar BCC microstructure.

The yield strength and compressive strength of the TaNbVTiAl_x_ RHEAs first increased and then decreased with the increase of Al content. Compression plasticity was decreasing. The Al0.2 RHEA had the highest yield strength and compressive strength. According to the traditional theory of solution strengthening [15], the yield strength should increase with the increase of Al content, but the actual situation was different. This shows that the traditional solid solution strengthening theory cannot fully explain the relationship between strength and solute Al. Moreover, Al atoms formed covalent bonds with other alloy atoms, which made the strengthening method more complex. 

In addition to solution strengthening, the effect of grain size should also be considered. Fine grains can produce fine grain strengthening benefits. With the increase of Al content, the grain size increased from 69 μm to 187 μm. This is one reason for the decrease in strength and plasticity. This change in compressive strength may have been caused by the change in lattice constant, which resulted from the addition of element Al. The higher the Al content, the smaller the lattice constant of TaNbVTiAl_x_ RHEAs. With the increase of Al content, the effect of covalent bond formed by the hybridization of p-layer saturated electron orbit and d-layer orbit of transition metal was more obvious. The covalent bond had a small length, which led to the decrease of the lattice constant. The change trend of the lattice constant was consistent with that of crystal surface spacing. As the Al content increased, the lattice constant decreased, so the interplanar spacing became smaller and the dislocation became more difficult to slip, making the yield strength increase [35,36]. When the Al content exceeded Al0.2, continuing to increase the Al content and lowering the interplanar spacing may have made the dislocations difficult to move, and thus generated dislocation accumulation and local stress concentration. When the stress concentration cannot be released, the alloy will break. This may be the main reason why the compressive strength and compression plasticity of TaNbVTiAl_x_ RHEAs decreased when the Al content exceeded Al0.2. These results are highly consistent with the as-cast TaNbVTiAl_x_ [15] and NbTiMoVAl_x_ [18] RHEAs.

## 5. Conclusions

1. The P/M TaNbVTiAl_x_ RHEAs showed a simple BCC microstructure with no obvious segregation. The average grain sizes were 69, 101, 106, 135, 147, and 187 μm for the Al0, Al0.2, Al0.4, Al0.6, Al0.8, and Al1.0 RHEAs, respectively.
2. The P/M TaNbVTiAl_x_ RHEAs showed excellent room-temperature specific strength (207.11 MPa*cm^3^/g) and high-temperature specific strength (88.37 MPa*cm^3^/g at 900 °C and 16.03 MPa*cm^3^/g at 1200 °C) with reasonable plasticity, suggesting that the material has the possibility for use at high temperatures > 1200 °C.
3. The reasons for the excellent mechanical properties of the P/M TaNbVTiAl_0.2_ RHEA were the uniform microstructure and solid solution strengthening effect.

## Figures and Tables

**Table 1:** The theoretical composition of the TaNbVTiAl_x_ refractory high-entropy alloys (RHEAs) (in at.%).

Alloys	Ta (at.%)	Nb (at.%)	V (at.%)	Ti (at.%)	Al (at.%)
Al0	25	25	25	25	0
Al0.2	23.81	23.81	23.81	23.81	4.76
Al0.4	22.73	22.73	22.73	22.73	9.09
Al0.6	21.74	21.74	21.74	21.74	13.04
Al0.8	20.83	20.83	20.83	20.83	16.67
Al1.0	20	20	20	20	20

**Table 2:** The characteristics of the raw powders.

Raw Powder	Average Particle Size (μm)	O (wt.%)	C (wt.%)	H (wt.%)
Ta	24.5	0.13	0.0064	0.0008
Nb	26.1	0.34	0.0220	0.0015
V	22.6	0.29	0.0096	0.0014
Ti	29.0	0.28	0.0150	0.0147
Al	18.6	0.27	0.0250	0.0017

**Table 3:** Crystal structures, calculated lattice parameters, theoretical lattice parameters, atomic radiuses, calculated melting temperatures, and theoretical densities of the TaNbVTiAl_x_ RHEAs.

	Ta	Nb	V	Ti	Al	Al0	Al0.2	Al0.4	Al0.6	Al0.8	Al1.0
Crystal structure	BCC	BCC	BCC	HCP	FCC	BCC	BCC	BCC	BCC	BCC	BCC
Calculated lattice parameter (Å)	-	-	-	-	-	3.243	3.236	3.228	3.227	3.213	3.210
Theoretical lattice parameter (Å)	3.303	3.301	3.039	3.276	4.050	3.230	3.269	3.304	3.337	3.366	3.394
Atomic radius (Å)	1.47	1.47	1.35	1.46	1.43	-	-	-	-	-	-
T_m_ (K)	3293	2750	2202	1946	933.5	2548	2471	2401	2337	2278	2225
ρ(g/cm^3^)	16.65	8.57	6.11	4.51	2.70	9.16	8.86	8.58	8.33	8.10	7.89

**Table 4:** Room-temperature compressive properties of the RHEAs in the references and the present work.

Materials	Density (g/cm^3^)	Preparation Methods	Phase Structure	Yield Strength (MPa)	Fracture Strength (MPa)	Plastic Strain	Specific Yield Strength (MPa*cm^3^/g)
NbMoTaW [3]	13.7	As-cast	BCC	1058	1211	1.5%	77.23
TaNbHfZr [23]	11.1	As-cast	BCC	1315	1885	21.6%	118.47
TiZrNbTa [19]	9.94	As-cast	BCC	1100	-	48%	110.66
TaNbHfZrTi [24]	9.9	As-cast	BCC	929	-	>50%	93.84
Al_0.4_Hf_0.6_NbTaTiZr [12]	9.05	As-cast	BCC	1841	2269	~5%	203.43
Al_0.21_HfNbTiZr [25]	8.12	As-cast	BCC	831	915.2	~20%	102.34
NbTiVTa [15]	9.16	As-cast	BCC	1092	-	>50%	119.21
NbTiVTaAl_0.25_ [15]	8.78	As-cast	BCC	1330	-	>50%	151.48
NbTiVTa_0.5_ [15]	8.45	As-cast	BCC	1012	-	>50%	119.76
NbTiVTa_1.0_ [15]	7.89	As-cast	BCC	991	-	>50%	125.60
TaNbVTi	9.16	PM	BCC	1391	1932	15%	151.86
TaNbVTiAl_0.2_	8.86	PM	BCC	1835	2217	10%	207.11
TaNbVTiAl_0.4_	8.58	PM	BCC	1719	2054	9%	200.35
TaNbVTiAl_0.6_	8.33	PM	BCC	1697	1810	5.5%	203.72
TaNbVTiAl_0.8_	8.10	PM	BCC	1606	1695	5%	198.27
TaNbVTiAl_1.0_	7.89	PM	BCC	1450	1619	2.5%	183.78

**Table 5:** The calculated values of ΔSmix, ΔHmix, Tm, Ω, valence electron concentration (VEC), and δ of the TaNbVTiAl_x_ RHEAs.

Alloys	ΔS_mix_ (J/K·mol)	ΔH_mix_ (kJ/mol)	T_m_(K)	Ω	VEC	δ (%)
Al0	11.53	−0.25	2548	117.51	4.75	3.53
Al0.2	12.57	−3.99	2471	7.78	4.67	3.44
Al0.4	13.01	−7.07	2401	4.42	4.59	3.37
Al0.6	13.24	−9.60	2337	3.22	4.52	3.29
Al0.8	13.35	−11.70	2278	2.60	4.46	3.23
Al1.0	13.38	−13.44	2225	2.22	4.40	3.16
